# Anger Is More Influential than Joy: Sentiment Correlation in Weibo

**DOI:** 10.1371/journal.pone.0110184

**Published:** 2014-10-15

**Authors:** Rui Fan, Jichang Zhao, Yan Chen, Ke Xu

**Affiliations:** 1 State Key Laboratory of Software Development Environment, Beihang University, Beijing, P. R. China; 2 School of Economics and Management, Beihang University, Beijing, P. R. China; Cinvestav-Merida, Mexico

## Abstract

Recent years have witnessed the tremendous growth of the online social media. In China, Weibo, a Twitter-like service, has attracted more than 500 million users in less than five years. Connected by online social ties, different users might share similar affective states. We find that the correlation of anger among users is significantly higher than that of joy. While the correlation of sadness is surprisingly low. Moreover, there is a stronger sentiment correlation between a pair of users if they share more interactions. And users with larger number of friends possess more significant sentiment correlation with their neighborhoods. Our findings could provide insights for modeling sentiment influence and propagation in online social networks.

## Introduction

From the view of conventional social theory, *homophily* leads to connections in social networks, as the saying “Birds of a feather flock together” states [1]. Even in the online social network, more and more evidence indicates that the users with similar properties would be connected in the future with high probabilities [2], [3]. It is clear that *homophily* could affect user behavior both online and offline [4], [5], while the records in online social networks are relatively easier to be tracked and collected. Moreover, the continuous growth of the online social media attracts a vast number of internet users and produces many huge social networks. Twitter(www.twitter.com), a microblogging website launched in 2006, has over 300 million active users, with over 500 million microblog posts, known as tweets, being posted everyday. In China, Weibo(www.weibo.com), a Twitter-like service launched in 2009, has accumulated more than 500 million registered users in less than five years. Every day there will be more than 100 million Chinese tweets published. The high-dimension content generated by millions of global users is a “big data” window [6] to investigate the online social networks. That is to say, these large-scale online social networks provide an unprecedented opportunity for the study of human behavior.

Beyond typical demographic features such as ages, races, hometowns, common friends and interests, *homophily* also includes psychological states, like loneliness and happiness [1], [4], [7]. Previous studies also show that the computer-mediated emotional communication is similar to the traditional face-to-face communication, which means there is no evident indication that human communication in online social media is less emotional or less personally [8]. Each user in the online social network could be a social sensor and the huge amount of tweets convey complicated signals about the users and the real-world events, among which the sentiments are an essential part. Therefore, emotion states of the users play a key role in understanding the user behaviors in social networks, whether from an individual or group perspective [9]–[11]. Meanwhile, users' mood states are significantly affected by the real-world events [12], which could be employed to predict the stock market [13] or to detect the abnormal event [14]. Recent study [4] shows that happiness is assortative in Twitter network and [6] finds that the average happiness scores are positively correlated between the Twitter users connected by one, two or three social ties. An interesting phenomenon of emotion synchronization is also unraveled in [15]. While in these studies, the human emotion is simplified to two classes of positive and negative or just a score of general happiness, neglecting the detailed aspects of human sentiment, especially the negative emotion. Because of oversimplification of the emotion classification, it is hard for the previous literature to disclose the different correlations of different sentiments and then make comparisons. However, the negative emotions, like anger, sadness or disgust, are more applicable in real world scenarios such as abnormal event detection or emergency tracking [14]. In [5], the authors also find that negative emotion could boost user activity in BBC forum. In fact, figuring out the correlation of these emotions might shed light on understanding why people participate in the diffusion of abnormal event in the network and how the large-scale collective behavior could form across the entire network. On the other hand, the investigation of how the local structure affects the emotion correlation is not systematically performed yet, while which is essential to studying the mechanism of sentiment influence and contagion.

Aiming at filling these vital gaps, we divide the sentiment of an individual into four categories, including *anger*, *joy*, *sadness* and *disgust*, and investigate the emotion correlation between connected users in the interaction network obtained from Weibo. Out of our expectation, it is found that *anger* has a stronger correlation between different users than that of *joy*, while *sadness*'s correlation is trivial. Further analysis demonstrates that *anger* in Weibo is related with the real-world events about food security, government bribery or demolition scandal, which are always the hot trends in Internet of China. Moreover, node degree, node clustering and tie strength all could positively boost the emotion correlation in online social networks, especially for the mood of *anger*. Finally, we make our data set in this paper publicly available to the research community.

## Materials and Methods

### Weibo Dataset

**Figure 1:**
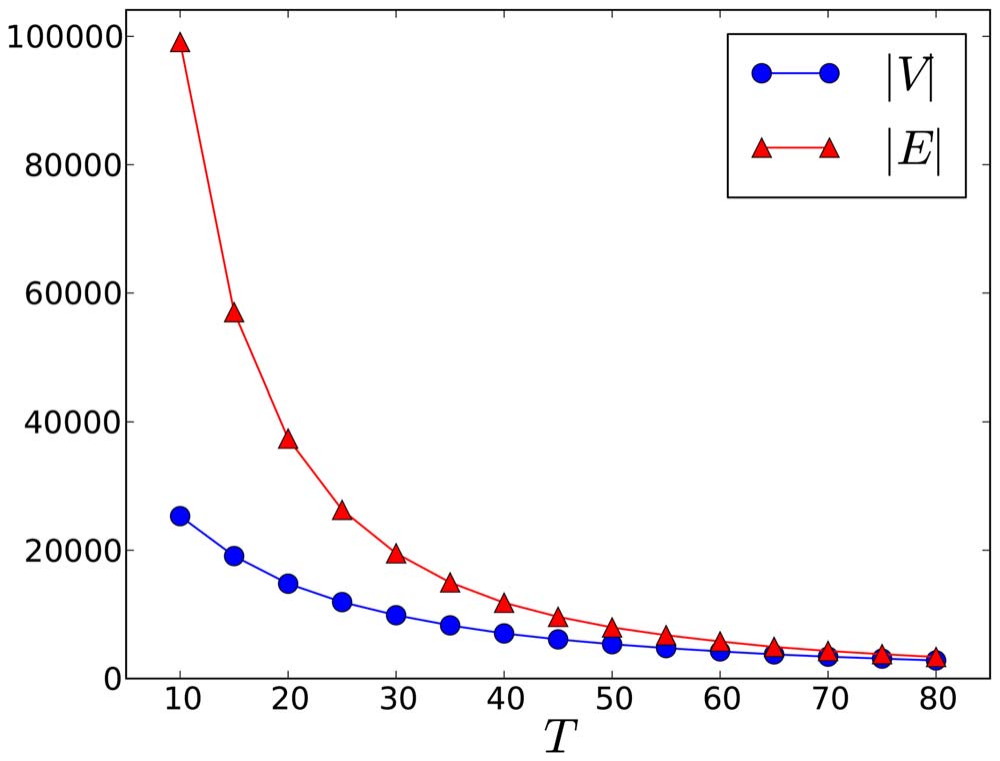
The number of nodes or edges varies for different interaction threshold 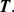 In the following part of the present work, we set 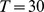 to extract a large enough network with convincing interaction strength.

As pointed out in [4], the following relationship in Twitter-like social networks does not stand for the social interaction, while if two users reply, retweet or mention each other in their tweets for certain times, the online social tie between them is sufficient to present an alternative means of deriving a conventional social network [6]. Starting from several influential seeds (like the users verified by Weibo), we adapt a typical Breadth-First-Search strategy to crawl tweets from Weibo through its open APIs. For each user we get, we first save all its tweets into the database and then add its followers (users that follow it) into the candidate queue for further explorations. Finally from December 2010 to February 2011, we accumulated around 70 million tweets posted by 278,654 users. While here we only construct an interaction network from the tweets posted during April 2010 to September 2010, where interaction means the number that two users retweet or mention each other is larger than a threshold 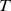. From around 26 million tweets posted during the period we select and 140,000 users we crawled, an undirected but weighted graph 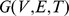 is constructed, in which 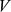 is the set of users (the ones without links are omitted), 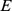 represents the set of interactive links among 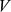, and 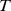 is the minimum number of interactions on each link. For each link in 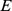, its weight is the sum of retweeting or mentioning times between its two ends in the specified time period. Specifically, to exclude occasional users that are not truly involved in the Weibo social network, we only reserve those active users in our interaction network that posted more than one tweet every two days on average over the six months. And to guarantee the validity of users' social interaction, if the number of two users retweet or mention each other is less than 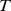, we would omit the connection between them. As shown in Figure 1, by tuning 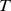 we can obtain networks of different scales. Generally we set 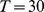 and then the interaction network 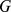 contains 9,868 nodes and 19,517 links. We also make our entire dataset publicly available(http://www.datatang.com/data/44650, http://goo.gl/iXzoXm). Note that here we collect tweets from Weibo through its open APIs(http://open.weibo.com) under the authority granted by Weibo and we have also anonymized user IDs and names in the published data set to protect users' privacy.

### Emotion classification

The content in online social media like Twitter or Weibo is mainly recorded in the form of short text. Many approaches have been presented to mine sentiment from these texts in recent years. One of them is the lexicon based method, in which the sentiment of a tweet is determined by counting the number of sentimental words, i.e., positive terms and negative terms. For example, Dodds and Danforth measured the happiness of songs, blogs and presidents [16]. They also employed Amazon Mechanical Turk to score over 10,000 unique English words on an integer scale from 1 to 9, where 1 represents sadness and 9 represents happiness [9]. Golder and Macy collected 509 million English tweets from 2.4 million users in Twitter, then measured the positive and negative affects using Linguistic Inquiry and Word Count(LIWC) (http://www.liwc.net). While another one is the machine learning based solution, in which different features are considered to perform the task of classification, including terms, smileys, emoticons and etc. The first step was taken by Pang et al. in [17], they treated the sentiment classification of movie reviews simply as a text categorization task and investigated several typical classification algorithms. According to the experimental results, machine learning based classifiers outperform the baseline method based on human words list [18]–[20]. Different from most work which just categorized the emotion into negative and positive, our previous work [14] divided the sentiment into four classes, then presented a framework based on emoticons without manually-labelled training tweets and achieved a convincing precision. Because of the ability of multi-emotions classification, we employ this framework in the present paper.

A vast number of training samples is necessary for handing the extremely short text in social media. To avoid intensive labor, we use emoticon to label tweets into different emotions. It has been found that both smiley and emoticon are strongly related with typical sentiment words and could be convincing indicators of different emotions [21]. They help the users to express their moods when post the tweet [22]. Tossell et al. also confirm that emoticon usage is contextual [23]. Hence, we could treat these emoticons as sentiment labels of the tweets. In fact, it is a kind of crowdsourcing, i.e., the users label the tweet with emoticons to express their emotions themselves [14]. In the labeling stage, first we manually label the emotion of the emoticon. We select the most popular 95 emoticons and several students are working separately to label their emotions. Their judgements are based on the image of the emoticon and around 50 frequent words occurring together with the emoticon. Finally we find that most of the emoticons are labelled by four sentiments, including *anger*, *joy*, *disgust* and *sadness*. For other emotions like *fear* or *surprise*, we do not find enough votes. So we split the emotion into these four classes. In fact it is also sort of consistent with the traditional Chinese culture that the human emotion are mainly constituted by four elements, including pleasure, anger, sorrow and joy.

From around 70 million tweets, 3.5 million tweets with valid emoticons are extracted and labeled. Using this data set as a training corpus 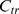, a simple but fast Bayesian classifier is built in the second stage to mine the sentiment of the tweets without emoticons, which are about 95% in Weibo. Be specific, for each tweet 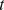 in 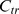, we converts it into a sequence of words 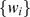, where 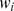 is a word and 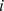 is its position in 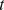. From the labeled tweets, we could obtain the word 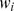
's prior probability of belonging to the sentiment category 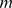 is 
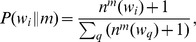
 where 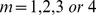, 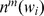 is the times that 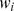 appears in all the tweets in the category 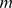 and *Laplace smoothing* is used to avoid the problem of zero probability. Then for an unlabeled tweet 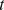 with word sequence 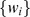, its category could be obtained as 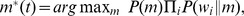 where 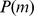 is the prior probability of 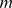. The averaged precision of this classifier is 64.3% and particularly the large amount of tweets we employ in the experiment can guarantee its accuracy further. For example, in the applications like *MoodLens*(http://gana.nlsde.buaa.edu.cn/hourly_happy/moodlens.html) and *Sentiment Search*(http://xinqings.nlsde.buaa.edu.cn/), it can be used to detect abnormal events effectively in real-time tracking. Moreover, the mechanism of incremental learning in this classifier can tackle the problems like sentiment drift of terms [9] or emergence of new features [14].

**Figure 2:**
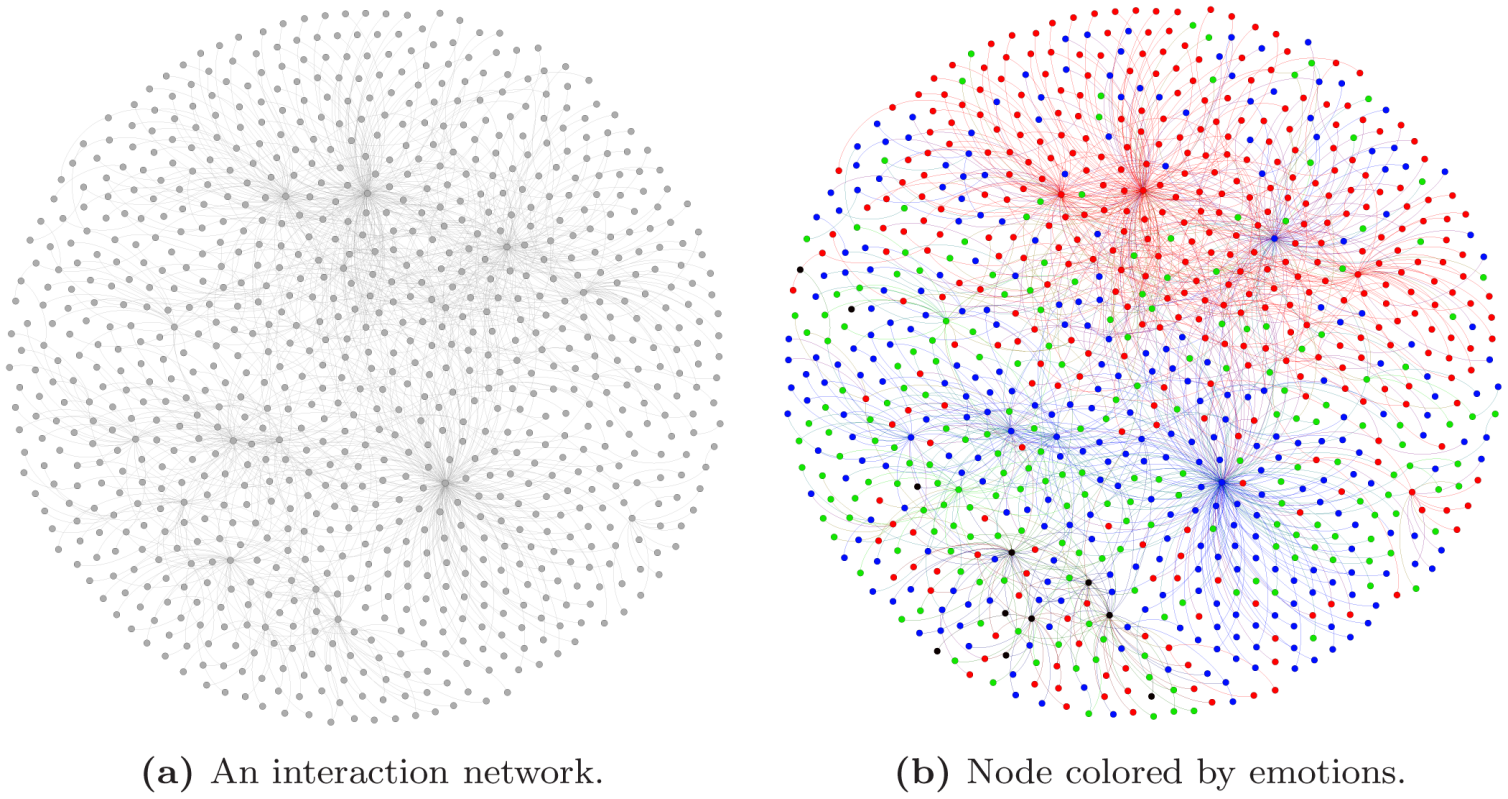
(Color online) The giant connected cluster of a network sample with 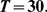 (a) is the network structure, in which each node stands for a user and the link between two users represents the interaction between them. Based on this topology, we color each node by its emotion, i.e., the sentiment with the maximum tweets published by this node in the sampling period. In (b), the red stands for *anger*, the green represents *joy*, the blue stands for *sadness* and the black represents *disgust*. The regions of same color indicate that closely connected nodes share the same sentiment.

Based on this framework, we demonstrate a sampled snapshot of the interaction network with 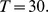 As shown in Figure 2b, in which each user is colored by its emotion. We can roughly find that closely connected nodes generally share the same color, indicating emotion correlations in Weibo network. Besides, different colors show different clusterings. For example, the color of red, which represents *anger*, shows more evident clustering. These preliminary findings inspire us that different emotions might have different correlations and a deep investigation is indeed necessary.

### Emotion correlation

Emotion correlation is a metric to quantify the strength of sentiment similarity between connected users. For a fixed 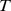, we first extract an interaction network 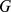 and all the tweets posted by the nodes in 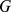. Then by employing the classifier established in the former section, the tweets for each user are divided into four categories, in which 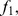 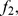 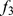 and 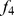 represent the fraction of angry, joyful, sad and disgusting tweets, respectively. Hence we can use emotion vector 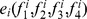 to denote user *i*'s sentiment status. Based on this, we define the pairwise sentiment correlation as follows. Given a certain hop distance 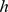, we collect all user pairs with distance 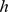 from 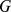. For one of the four emotions 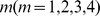 and a user pair 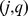, we put the source user *j*'s 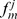 into a sequence 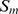, and the target user *q*'s 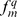 to another sequence 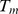. Then the pairwise correlation could be calculated by Pearson correlation as
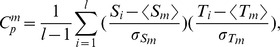
where 
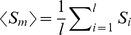
 is the mean, 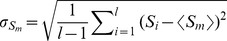 is the standard deviation and 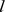 is the length of 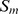 or 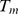. Or it can also be obtained from Spearman correlation as
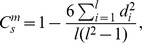
where 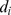 is the rank difference between 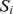 in 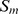 and 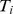 in 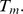 Intuitively larger 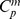 and 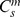 both suggest a more positive correlation for sentiment 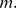 In order to investigate fluctuations in the sentiment correlation, we also use the approach of *bootstrap* [24] to perform the error analysis. For instance, given two emotion sequences of length 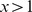, denoted as 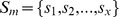 and 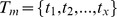, we do not calculate their correlation directly. Contrarily, we first uniformly sample 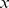 integer indexes from the range of 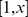 with replacement and then put them into an index sequence defined as 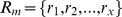. Through traversing each index 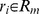, we can construct two new lists by putting 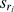 into 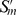 and 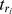 into 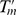, respectively. Obviously after this we can generate two sampled sequences 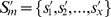 and 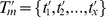. Finally for each round of index-sampling we can obtain a correlation value between 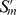 and 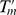, and through 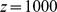 times of repetitions we would obtain an averaged correlation and a standard deviation (the error) for *m*. Apparently lower errors stand for more significant correlations. Note that in the rest of the paper, averaged correlation would be presented as correlation if there is no conflict in the context.

Based on the dataset and classifier, interaction networks could be built and tweets of each user in the network would be emotionally labelled. Using the definition of correlations, we can then present the comparison of emotion correlations and the impact of local structures in the following section.

## Results

**Figure 3:**
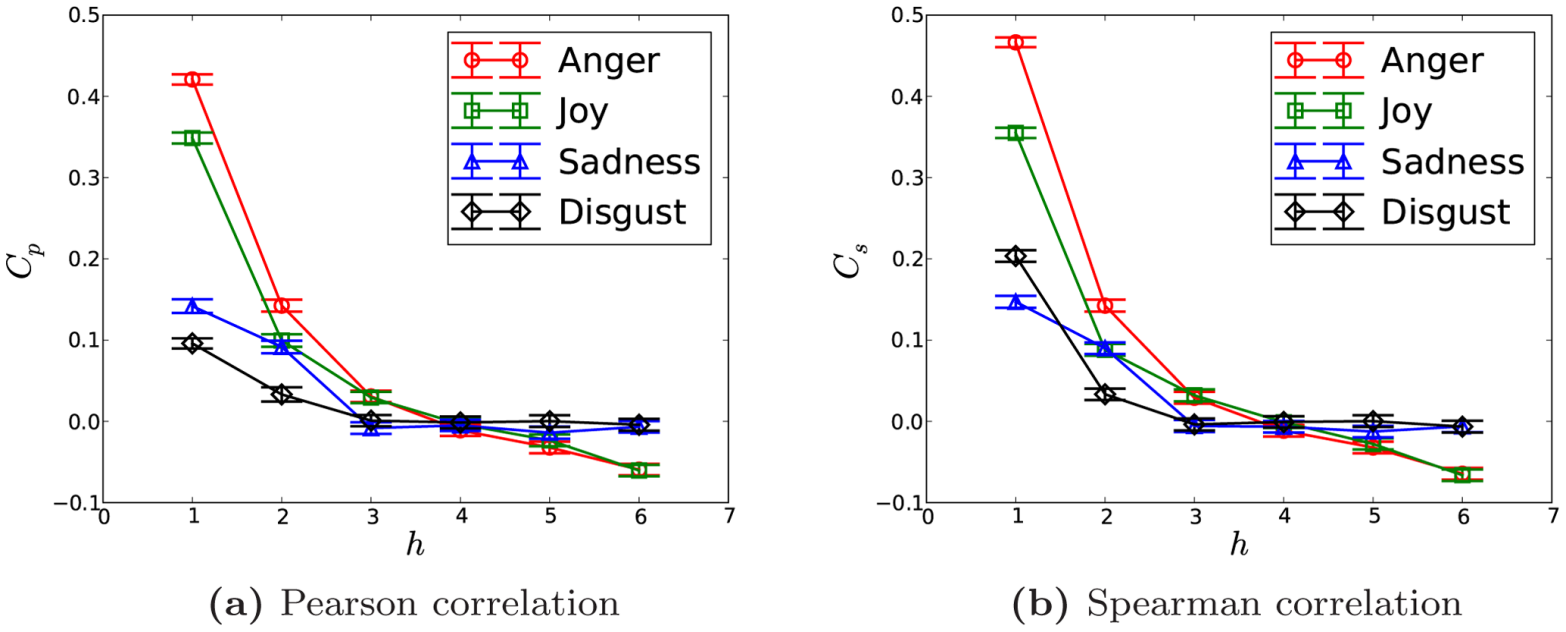
Correlations with error-bar for different emotions as the hop distance varies. Large 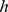 means a pair of users are far away from each other in the social network we build. Here 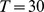 is fixed.

First we compare the correlation of different emotions based on the graph of 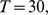 which ensures enough number of ties and users, and at the same time guarantees relatively strong social tie strength. As shown in Figure 3, both Pearson correlation and Spearman correlation indicate that different sentiments have different correlations and *anger* has a surprisingly higher correlation than other emotions. In addition, the standard deviations of all sentiments' correlations are extremely small, which indicates that the sentiment correlation in online social networks is indeed significant and only shows trivial fluctuations. Although the previous studies [4], [6] show that happiness is assortative in online social networks, but Figure 3 further demonstrates that the correlation of *anger* is much stronger than that of happiness, especially as 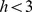. While for *sadness* and *disgust*, they both have an unexpected low correlation even for small 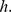 For instance, the correlation of *sadness* is less than 0.15 as 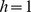. The results are also consistent with the previous findings that strength of the emotion correlation decreases as 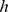 grows, especially after 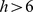 [6]. In fact, as 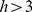, the emotion correlation becomes weak for all the sentiments, which means that the correlation of the sentiment in the social network is limited significantly by the social distance. For example, for strong assortative emotions like *anger* and *joy*, their correlations just fluctuate around 0 as 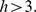


**Figure 4:**
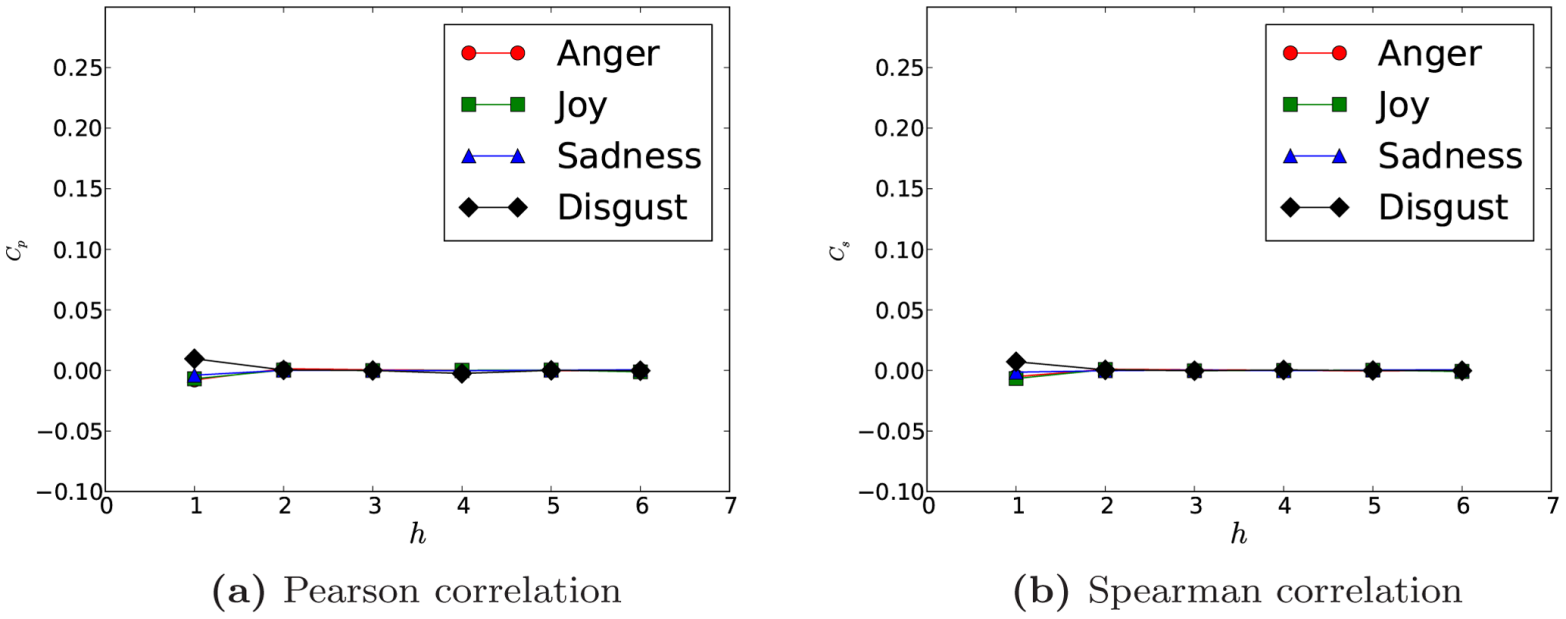
The emotion sequence is randomly shuffled to test the correlation significance.

In order to test the above correlation further, we also shuffle 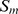 and 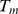 randomly for sentiment 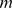 and recalculate its correlation. As shown in Figure 4, for the shuffled emotion sequence, there is no correlation existing for all the sentiments. It indicates that the former correlation we get is truly significant and for random pairs of users in the social network, there is no *emotion homophily*. It further justifies that through social ties, closely connected friends indeed share similar affective states.

**Figure 5:**
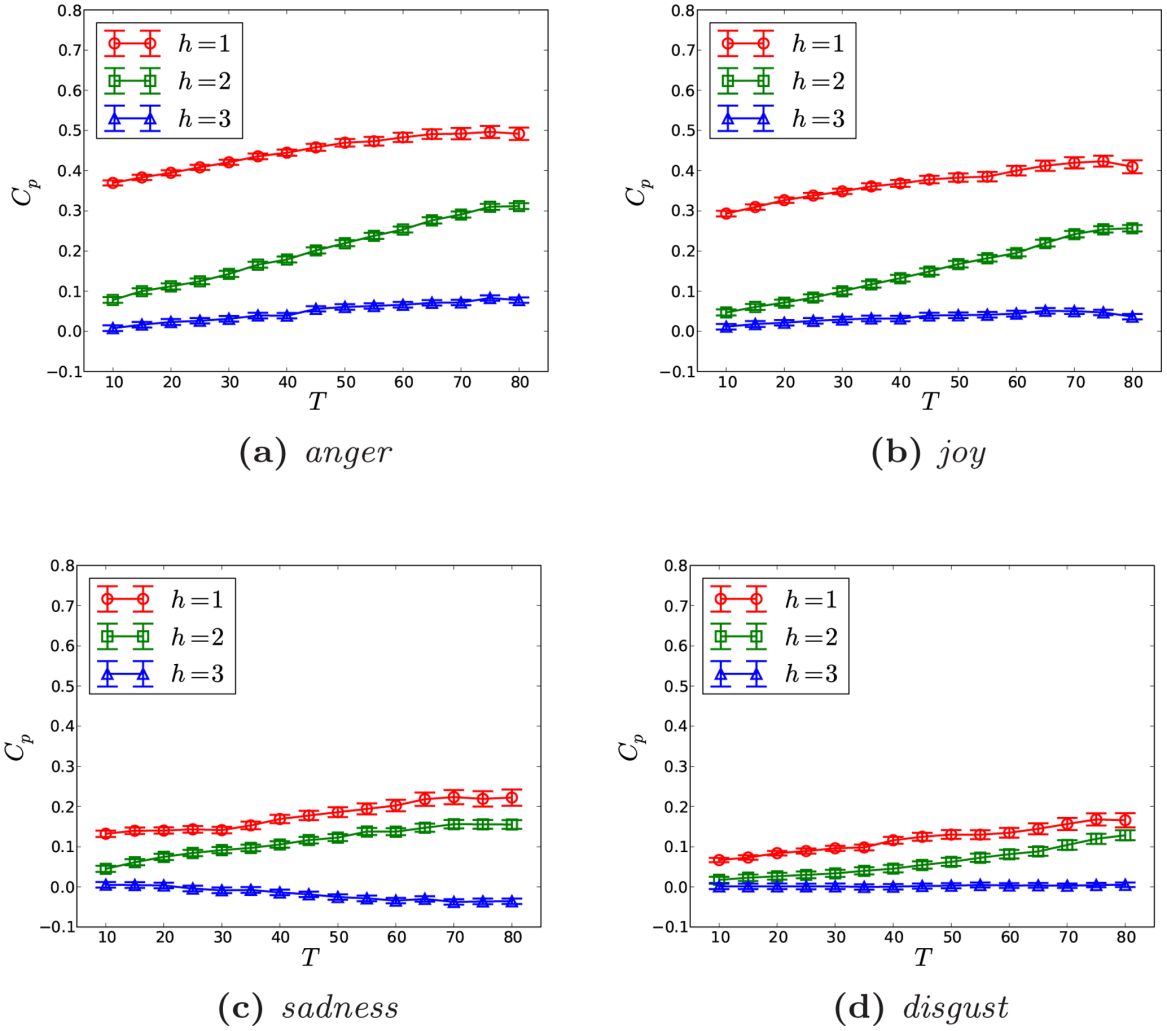
Pearson correlations of different 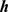 for different networks extracted by varying 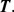 The case of 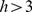 is not considered here because of the weak sentiment correlation found in Figure 3.

**Figure 6:**
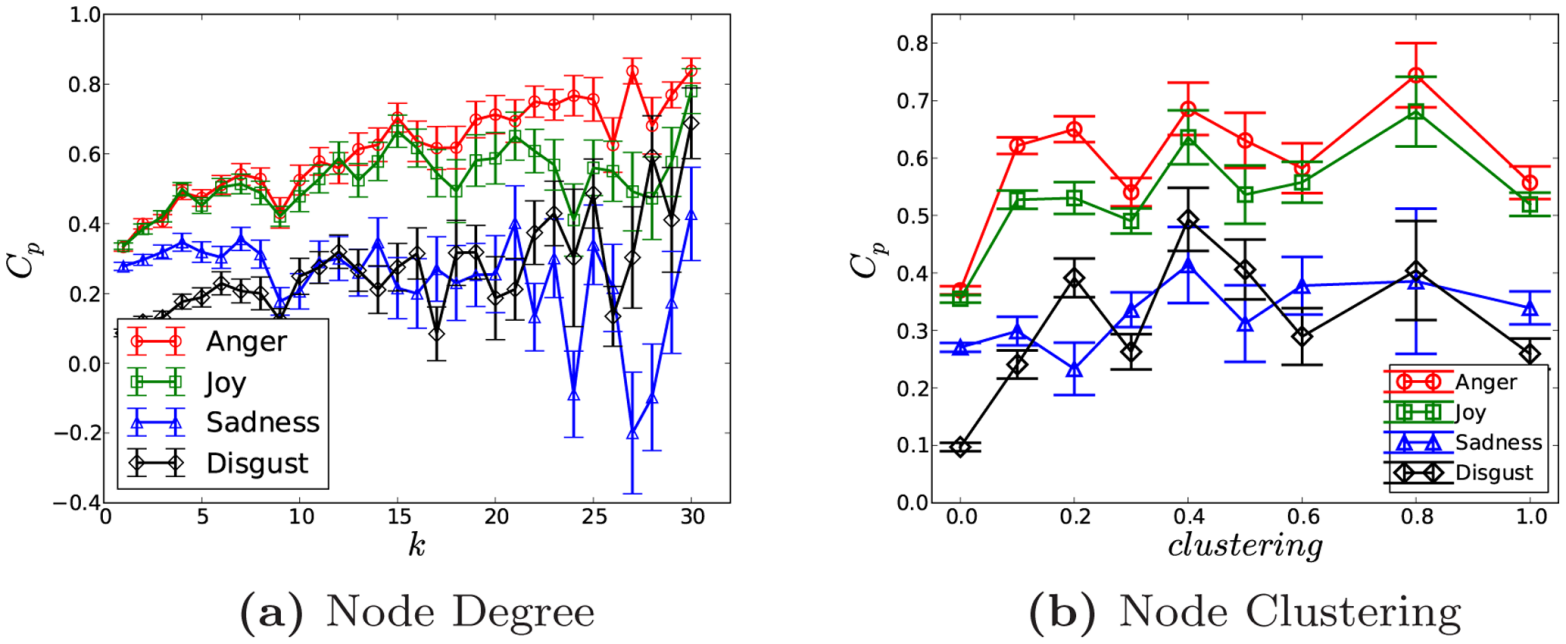
Here 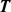 is fixed to 10 to reduce the data sparsity. Because the network is relatively small, the largest degree we get is only 30. Therefore, the results in Figure 6a just demonstrate that when the degree is small, how the sentiments' correlations vary with node degrees. While regarding to Figure 6b, the linear bin is used to get emotion sequences for nodes with clusterings within the same bin.

Investigating to what extent the local structure, like tie strength, node degree and node clustering, could affect the emotion correlation and its error is of importance for modeling sentiment influence and propagation in future work. As shown in Figure 5, we first disclose how the interaction threshold 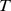 affects the sentiment correlations. As discussed in Section *Weibo Dataset*, larger 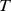 produces smaller networks but with closer social relations and more frequent online interactions. It is also intuitive that frequent interactions in online social networks are positively related with strong social ties and convincing social bonds. Because of this, we can see in Figure 5 that for all the four emotions, their correlations inside two hops continue a steady increasing trend with 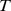
's growth. Particularly for *anger*, its Pearson correlation could rise to around 0.5. For weakly correlated emotions like *sadness* and *disgust*, although the correlation shows a slow growth for 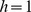 and 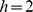, while the maximum value of the correlation is still lower than 0.25. As 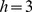, the increment of the sentiment correlation is trivial, especially for *sadness* and *disgust*. It illustrates that the primary factor of controlling the emotion correlation is still the social distance and the social tie strength just functions for close neighbors in the scope of two hops. Note that as 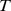 grows, the size of the network is reduced and the length of emotion sequences would be shortened accordingly, which might import more noise and produce larger errors. As can be seen in Figure 5, errors of correlations grow with 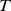, especially as 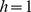. Secondly, we check the effect of users' degrees to the sentiment correlation. Given a random node 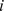 with degree 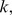 we have a sequence of the number of tweets with sentiment 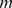 for its friends, which is denoted as 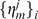 and 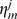 is the number of tweets with emotion 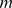 posted by an arbitrary neighbor 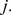 Then the number of tweets with emotion 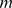 posted by 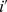
s neighborhood is 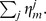 Ultimately the sentiment vector for 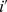
s neighborhood could be defined as 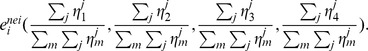 Through adding 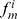 into 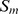 and 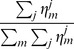 into 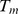, we could get the correlation of sentiment 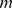 for the users with degree 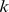. As can be seen in Figure 6a, the sentiment correlation grows with 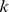, especially for *anger* and *joy*, which illustrates that nodes with higher degrees in online social networks possess stronger emotion correlation with their neighborhoods. That is to say, having more friends in the social media indicates more significant sentimental correlation with the neighborhood. Specifically, the correlation of *anger* and *joy* are almost same for very small degrees, but later *anger* shows a significant jump for large degrees and enlarges the gap as compared to *joy*. As 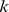 raises to 30, the correlation of *anger* grows almost to 0.85. While the correlation of *sadness* and *disgust* do not demonstrate an obvious increasing trend and just fluctuate around 0.2 or even lower. Similarly, here errors also grow with increasing 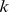, because nodes with large degrees only occupy a little fraction in the online social network [25], which would reduce the length of the emotion sequence and import noise, especially for *sadness* and *disgust* as shown in Figure 6a. However, *anger*'s correlation is still significantly higher than that of *joy* as 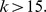 It is also worthy emphasizing that because the network size is small and we only have the maximum degree around 30, which is far below the Dunbar's Number [25], [26]. We suspect that the correlation might stop rising if the degree is larger than Dunbar's Number. Thirdly, we investigate the impact of the clustering of a node 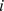, which is defined as 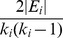, where 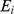 is the set of ties among *i*'s friends and 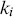 is the degree of *i*. For 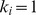, we set *i*'s clustering to zero. Then similar to the case of degree, we could get the correlation variation as clustering grows for different emotions. As shown in Figure 6b, correlations of *anger* and *joy* grow very slowly with the clustering, while *disgust* and *sadness* just demonstrate fluctuations without obvious increment. The correlation of *anger* is still stronger than that of *joy*. However, different from the case of degree, even the correlations of *anger* and *joy* fluctuate as the clustering rises. While with respect to the error, it again shows a rising trend with the growth of clustering, since nodes with highly clustered neighborhoods take a trivial fraction in the social network [25]. Generally the above observations indicate that for *anger* and *joy*, the emotion correlation between a node and its neighborhood would be a little bit stronger as its neighbors are more closely clustered. The results of Spearman correlation are similar and not reported here.

To sum up, different emotions have different correlations in the social media. Compared to other sentiments, *anger* has the most positive correlation. Local structures can affect the sentiment correlation in near neighborhoods, from which we can learn that tie strength, node degree and node clustering could enhance the sentiment correlation, especially for *anger* and *joy*, and their contributions to *sadness* and *disgust* are greatly limited.

## Discussion

Users with similar demographics have high probabilities to get connected in both online and offline social networks. Recent studies reveal that even the psychological states like happiness are assortative, which means the happiness or well-being is strongly correlated between connected users in online social media like Twitter. Considering the oversimplification of the sentiment classification in the previous literature, we divide the emotion into four categories and discuss their different correlations in details based on the tweets collected from Weibo of China, and the dataset has been publicly available to research community. Our results show that *anger* is more significantly correlated than other emotions like *joy*. While out of our expectation, the correlation of *sadness* is low.

**Figure 7:**
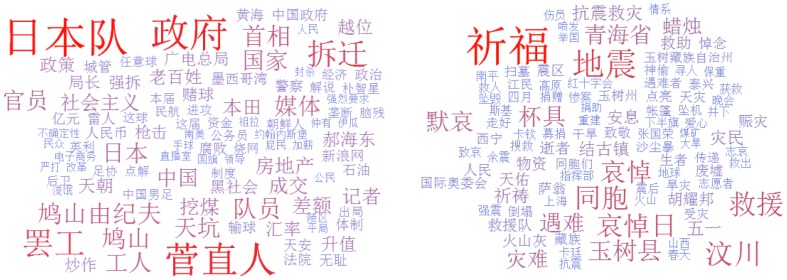
The example Chinese keywords extracted for *anger*(left) and *sadness*(right), respectively. The top 20 keywords are also translated into English, which could be found through http://goo.gl/tl4q45.

We try to unravel the underlying reason of why *anger* has a surprisingly high correlation but the correlation of *sadness* is weak from the view of keywords the corresponding tweets present. For a certain emotion 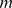, we collect all the retweeted tweets(usually contain phrase like “@” or “retweet”) with this sentiment in a specified time period to combine into a long text document. Focusing only on retweeted tweets could help reduce the impact of external media and just consider the contagion from the social ties in Weibo. Several typical techniques are employed to mine the keywords or topic phrases from the documents [27], which are reported in Figure 7. Based on the keywords or topics we find, the real-world events or social issues could be summarized to understand the sentiment correlation in detail.

With respect to *anger*, we find two kinds of social events are apt to trigger the angry mood of users in Weibo. First one is the domestic social problems like food security, government bribery and demolition for resettlement. The “shrimp washing powder” which results in muscle degeneration and the self-burning event in Fenggang Yihuang County of Jiangxi province represent this category. These events reflect that people living in China are dissatisfied about some aspects of the current society and this type of event can spread quickly as the users want to show their sympathy to the victims by retweeting tweets and criticizing the criminals or the government. Frequently appearing phrases like “government”, “bribery”, “demolition” and so on are strongly related with these events. The second type is about the diplomatic issues, such as the conflict between China and foreign countries. For instances, in August 2010, United States and South Korea held a drill on the Yellow Sea, which locates in the east of China. In September 2010, the ship collision of China and Japan also made users in Weibo extremely rageful. Actually, these events could arouse patriotism and stimulate the angry mood. Keywords like “Diaoyu Island”, “ship collision” and “Philippines” show the popularity of these events at that time. To sum up, Weibo is a convenient and ubiquitously channel for Chinese to share their concern about the continuous social problems and diplomatic issues. Pushed by the real-world events, these users tend to retweet tweets, express their anger and hope to get resonance from neighborhoods in online social networks. While regarding to *sadness*, we find its strength of correlation is strongly affected by the real-world natural disasters like earthquake, as shown in Figure 7(right). Because the natural disaster happens occasionally and then the averaged correlation of the sadness is very low and the strength of its correlation might be highly fluctuated.

With the continuous growth, online social media in China like Weibo have been becoming the primary channel of information exchange. In Weibo, the messages do not only deliver the factual information but also propagate the users' opinions about the social event or individual affairs. Real-world society issues are easy to get attention from the public and people tend to express their feelings towards these issues through posting and retweeting tweets in online social media. Through keywords and topics mining in retweeted angry tweets, we find the public opinion towards social problems and diplomatic issues are always angry and this extreme mental status also possesses the strongest correlation between connected users in Weibo. We conjecture that *anger* plays a non-ignorable role in massive propagations of the negative news about the society, which are always hot trends in today's Internet of China. This might be the origin of large scale online collective behavior in Weibo about society problems such as food security and demolition for resettlement in recent years. It is also consistent with a finding that good news never goes beyond the gate while bad news spread far and wide by ancient Chinese people more than one thousand years ago [28]. It should also be mentioned that the study of this paper has received great attention from many media like MIT Technology Review, CNN, BBC and the Washington Post (just to name a few of them) after a preprint of this paper was posted on the Internet [29]. It is widely believed that similar results might also exist in the social media of other countries. In the future work we would investigate the role of emotion in the information diffusion and comprehensively understand how different sentiments function in the formation of the public opinion or the massive collective behavior. Meanwhile, our findings could inspire the modeling of emotion contagion, like different emotions might diffuse with diverse strengths, local structures such as tie strengths, degrees and clusterings might affect the spread and the emotion might only function between individuals with social distance no more than three hops. Another interesting direction is to study how to make the social media more neutral by introducing some new mechanisms, e.g. delaying the post of an angry tweet can give people additional time for consideration and so might be of use for reducing the number of angry tweets.
